# Table tennis musculoskeletal injuries in young high-level players: a 17-year retrospective cohort study

**DOI:** 10.1097/MS9.0000000000005269

**Published:** 2026-07-10

**Authors:** Caroline Bonhomme, Pierre Menu, Jérôme Grondin, Thomas Hirardot, Marc Dauty, Alban Fouasson-Chailloux

**Affiliations:** a Service de Médecine Physique et Réadaptation Locomotrice, CHU Nantes, Nantes Université, Nantes, France; b Service de Rhumatologie, CHU Nantes, Nantes Université, Nantes, France; c INSERM UMR U1229/RMeS, Regenerative Medicine and Skeleton—Nantes Université, Nantes, France; d Institut Régional de Médecine du Sport (IRMS), Nantes, France

**Keywords:** cohort study, injury, knee, osteochondrosis, overuse, racket sport, sport injuries

## Abstract

**Background:**

Table tennis is a racket sport that has been growing in popularity in recent years. The current scientific literature on injuries remains limited. We aimed to assess the incidence and nature of injuries in young table tennis players.

**Methods:**

We conducted a 17-year retrospective single-center cohort study in the Department of Sports Medicine at a University Hospital. One hundred and thirty-nine national-level table tennis athletes aged 13.3 ± 2.1 years were studied. The cohort was divided into four groups according to age categories (U11: 8–11 years, U13: 12–13 years, U15: 14–15 years, and U19: 16–19 years). The main outcome measure was the injury rate per season and per athlete.

**Results:**

The injury rate was 1.2 injuries per 1000 hours of exposure and 0.7 injuries per season per athlete. There was no significant difference in injury rate between age categories (*P* = 0.57). The most frequently injured body parts were knees (30.5%), feet (16.8%), lumbar spine (14.7%), shoulders (12.6%), and groins (10.5%). Seventy-six injuries (82.6%) were overuse injuries. The most common diagnosis was growth-related injuries (40%); these primarily affected the knee (15.8% had Osgood–Schlatter disease and 9.5% had Sinding–Larsen disease), followed by the foot (13.7% had Sever’s disease). The next most frequently represented conditions were shoulder tendinopathies (11.6%), chronic low back pain (8.4%), and hip impingement (6.3%).

**Conclusion:**

Most of the injuries were chronic overuse injuries. A thorough understanding of the nature of injuries and their mechanisms of occurrence is essential to improving medical management as well as implementing effective preventive measures.

## Introduction

Table tennis is a racket sport that has been growing in popularity in recent years. In France, table tennis has been receiving increased media coverage along with a rise in the number of participants, which can be partially explained by the strong performances of some French players on the international stage. In 2024, the French Table Tennis Federation (FFTT) had more than 210 000 licensed players, representing an increase of over 15% in just 2 years^[^1^]^.

Table tennis has numerous positive effects on global health, particularly among young players, including improvement in gross motor skills, attention, coordination capacity, and cardiovascular benefits^[^2^]^. However, it is a complex sport that requires dynamic and explosive capacities and involves rapid movements of the lower limbs, trunk, and upper limbs, leading to a significant risk of musculoskeletal injuries for players^[^3^]^. The athletes must simultaneously analyze the game, react, move, and position themselves correctly to strike the ball under optimal conditions, thanks to the coordination of both upper and lower limbs, trunk rotation, and precise hand and footwork^[^4^]^. Each stroke demands fast and powerful movements, allowing the ball to reach speeds exceeding 120 km/h with more than 140 revolutions per second^[^5^]^.

HIGHLIGHTS

- The overall incidence rate in young table tennis players was 1.2 injuries per 1000 hours of exposure and 0.7 injuries per season per athlete.
- They mostly presented with chronic overuse injuries, mainly osteochondroses.
- The majority of injuries were located in the lower limbs (71.6%).
- A thorough understanding of the nature of injuries and their mechanisms of occurrence is essential to improving medical management and proposing effective preventive measures.

The current scientific literature on table tennis injuries remains limited. Studies conducted on international competitions report a relatively low incidence, with a prevalence of musculoskeletal injuries of 3.6% and an incidence of 10 injuries per 1000 hours of exposure^[^6^]^. On the other hand, national studies conducted over a longer period and including training sessions suggest a higher prevalence, with an incidence ranging from 2.8% to 44% and a predominance of micro-traumatic and overuse injuries^[^7^]^. Nevertheless, there is a lack of detailed data on etiologies and risk factors. Most of the studies have a moderate to high rate of bias, and very few studies have examined the epidemiology of injuries in young table tennis athletes^[^8,9^]^. However, this population is particularly at risk due to intensive training, a high number of practice hours, and significant physical demands. These constraints should also be balanced with academic requirements during a critical period of growth and development. Yet, the identification of injuries provides a better understanding of their mechanisms to implement appropriate preventive measures. Thus, to improve knowledge about injuries, we conducted a retrospective epidemiological cohort study among young table tennis athletes playing in a regional training center whose goal was to compete at the highest national or even international level. This retrospective cohort study has been reported in line with the STROCSS guidelines^[^10^]^.

## Materials and methods

We performed a descriptive, epidemiological retrospective cohort study among all young table tennis athletes followed at the Regional Institute of Sport Medicine of Nantes University Hospital from 1 January 2008 to 30 June 2025, covering a period of 17 years.

1. Population

Athletes were members of the “Pôle Espoir Tennis de Table de Loire Atlantique” and the “Structure Régionale Associée (SRA),” a regional training center and its recruiting structure. All the players were competing at the national level. The cohort was divided into four age groups according to the competition categories (from 8 to 11 years old, called “U-11”; 12–13 years old, called “U-13”; 14–15 years old, called “U-15 or cadets”; and from 16 to 19 years old, called “U-19 or Juniors”). We excluded those who did not belong to any of these categories.

2. Follow-up at the sports medicine department

The athletes received a mandatory annual medical check-up at the Sports Medicine department, as well as additional consultations in case of injuries. They also had a weekly visit to the training center by the team doctor, who was part of the Sports Medicine department at Nantes University Hospital, to address any trauma-related issues. During the annual medical check-up, their personal and family history, the number of training hours, the functional signs during exercise, and the history of injuries during the sports season were assessed. A general medical examination focusing on cardiopulmonary and musculoskeletal health was carried out in accordance with the FFTT practitioner’s form and based on any potential complaints. A morphological assessment with anthropometric measurements [weight, height, and body mass index (BMI)] as well as an electrocardiogram were performed by a nurse. The athletes also benefited from specialized consultations with a dietitian and a psychologist, aiming to identify nutritional and hydration errors, evaluate stress and anxiety levels, and detect symptoms of overtraining. The data were collected from the table tennis players’ medical records, kept by the team doctor and the physicians of the Regional Institute of Sport Medicine.

3. Definition of injury

We used the International Olympic Committee consensus statement: *methods for recording and reporting of epidemiological data on injury in sport 2020* for this study^[^11^]^. The injuries were defined as a physical complaint reported by an athlete about an injury occurring during the practice of table tennis in competition or training and requiring medical attention. The clinical and paraclinical elements that led to the diagnosis, as well as the nature, location, and severity of the injury, were sought in the patients’ medical files. The severity was measured by the duration of time loss, that is to say, the number of days that the athlete was unavailable for training and competing. Injuries were considered major if they resulted in more than 28 days off from sport. Mode of onset was classified as sudden (or acute) if the injury resulted from a specific identifiable event, or as gradual (the term “overuse injury” is also commonly used), related to overuse and repetitive movements. In cases of acute onset, the mechanism was further classified as either non-contact or contact. Contact was then categorized as contact with another player or with an object.

4. Diagnosis of injury

Diagnoses were assessed by experienced physicians from the Sports Medicine department at Nantes University Hospital. The prescription of medical imaging was left to the discretion of the examining physician and depended on the nature, severity, and evolution of the injury. The diagnosis was recorded in as much detail as possible, given the information available. Injuries were described according to the affected body area, the type of tissue involved, and the pathology, using the coding systems Sport Medicine Diagnostic Coding System and the Orchard Sports Injury Classification System^[^12^]^.

5. Exposure and incidence of injury

The exposure was estimated based on the number of training hours per week multiplied by the number of weeks of practice throughout the year. In addition, a 5-day training camp during school holidays (excluding the Christmas break) was included. We did not take into account the number of hours played in competition, as we did not have sufficiently reliable data on this aspect. Injury incidence was expressed per 1000 hours of table tennis practice, as well as incidence among all athletes followed up and among all injured athletes.

6. Statistical analysis

Data were recorded in Microsoft Excel 2016 (Microsoft Corporation, USA). Statistical analyses were performed using IBM SPSS 27.0 software (Armonk, NY, USA). Quantitative variables were expressed as mean and standard deviation (SD), while qualitative variables were expressed as numbers and percentages. We used the non-parametric Kruskal–Wallis test by ranks and the Dunn post-hoc test to compare quantitative variables. The Chi-squared (χ^2^) test was used to compare qualitative variables. The alpha level of significance was set at 0.05. Missing data were excluded from both the descriptive and comparative analyses.

7. Ethics

The study was approved by the Nantes Ethics Committee for Sports Medicine (CNEMS), approval number CNEMS-002-2025. All participants and their legal representatives signed a written consent form for the anonymous use of their data, and the study was conducted in compliance with the principles of the Declaration of Helsinki, as well as the ethical standards for research in sports and exercise science^[^13^]^. We declare no conflicts of interest related to the content of this study, nor any funding.

## Results

1. Patients’ characteristics

Over the 17 sports seasons, 139 table tennis athletes aged 13.3 ± 2.1 years were followed in the Sports Medicine department (Table 1). Thirty-three athletes were in the U-11 category (8–11 years old), 40 were in the U-13 category (12–13 years old), 48 were in the U-15 category (14–15 years old), and 18 were in the U-19 category (16–19 years old). There were 37.4% women, with no significant difference between categories (*P* = 0.89). The average number of hours of table tennis training per week was 14.3 ± 4.2 hours. The athletes in the U-11 category had significantly fewer training hours than those in the U-18 category (*P* = 0.039). The mean BMI was 19.2 ± 2.5 kg/m^2^, with a minimum of 15.2 kg/m^2^ and a maximum of 23.8 kg/m^2^. There was a significant difference in BMI between each category (*P* < 0.0001), except between the U-11 and the U-13 categories. The BMI increased with age categories.

2. Incidence of injury

**Table 1:** Population characteristics according to age level.

	Total *N* = 139	U-11 *N* = 33	U-13 *N* = 40	U-15 *N* = 48	U-19 *N* = 18	*P*
Age (year), mean ± SD	13.3 ± 2.1	10.52 ± 0.8 ^∬***, ∭***,***^	12.6 ± 0.5 ^∫***,∭***,†***^	14.5 ± 0.5 ^∫***,∬***,†*^	16.8 ± 1.1 ^∫***,∬***,∭*^	<0.0001
Sex: males/females, *n* (%)	87/52 (37.4)	22/11 (33.3)	25/15 (37.5)	30/18 (37.5)	10/8 (44.4)	0.89
Number of training hours per week, mean ± SD	14.3 ± 4.2	12.5 ± 2.8^†*^	15.0 ± 3.4	14.3 ± 3.5	17.3 ± 7.1^∫*^	0.039
Weight (kg), mean ± SD	49.0 ± 12.4	36.1 ± 6.8 ^∭***,†***^	43.9 ± 8.6 ^∭***,†***^	55.7 ± 7.8 ^∫***,∬***^	64.8 ± 6.0 ^∫***,∬***^	<0.0001
Height (cm), mean ± SD	159.0 ± 12.9	144.2 ± 8.2 ^∬*, ∭***^^, †***^	155.4 ± 8.5 ^∫*, ∭***,†***^	167.2 ± 8.3 ^∫***, ∬***^	171.0 ± 7.7 ^∫***, ∬***^	<0.0001
BMI (kg/m^2^), mean ± SD	19.2 ± 2.5	17.2 ± 2.0 ^∭***,†***^	18.0 ± 2.0 ^∭**,†***^	19.9 ± 2.0 ^∫***,∬**,†*^	22.2 ± 1.7 ^∫***, ∬***,∭*^	<0.0001

SD, standard deviation; Dunn post-hoc test: ^∫^ Significant difference with U-11, ^∬^ Significant difference with U-13, ^∭^ Significant difference U-15, and ^†^Significant difference U-19. *P ≤ 0.05; **P ≤ 0.01; ***P ≤ 0.001.

The 139 athletes followed over 17 years accumulated 76 541 hours of training. During the follow-up, 95 injuries were reported, representing an overall incidence rate of 1.2 injuries per 1000 hours of exposure and 0.7 injuries per season per athlete. There was no significant difference in injury rates between the different age categories (*P* = 0.57). Some athletes experienced multiple injuries during the season, with an incidence rate of 1.3 injuries per injured athlete. Thirty-four injuries (35.8%) resulted in time loss (Table 2). There was no significant difference based on age categories and the duration of time off from sport (*P* = 0.93).

3. Location of injuries and diagnoses

**Table 2:** Injuries characteristics according to age level.

	Total *N* = 139	U-11 *N* = 33	U-13 *N* = 40	U-15 *N* = 48	U-19 *N* = 18	P
Injuries/athletes, *n*	0.7	0.4	0.8	0.8	0.7	0.57
Injuries/injured athletes, *n*	1.3	1	1.3	1.3	1.3	0.91
Mode of onset ᵃ						
* Gradual, n (%)*	76 (82.6)	11	24	30	11	0.73
* Sudden, n (%)*	16 (17.4)	2	7	6	1	
Time loss injuries, *n* (%) ᵇ	34 (35.8)	6	12	13	3	
≤7 days	10	1	4	5	0	0.93
8–28 days	12	2	4	4	2	
*>*days	12	3	4	4	1	
Location						
* Upper limbs, n (%)*	13 (13.7)	0 (0)	1 (3.2)	9 (24.3)	3 (23.1)	0.06
* Lower limbs, n (%)*	68 (71.6)	13 (92.9)	24 (77.4)	24 (64.9)	7 (53.8)	
* Spine, n (%)*	14 (14.7)	1 (7.1)	6 (19.4)	4 (10.8)	3 (23.1)	

ᵃ Missing data: 3, ᵇ Missing data: 9.

The majority of injuries were located in the lower limbs (*n* = 68, 71.6%), followed by the spine (*n* = 14, 14.7%) and the upper limbs (*n* = 13, 13.7%) in relatively similar proportions. There was no significant difference in injury locations between the different age categories (*P* = 0.06). The most frequently injured body parts were the knees (30.5%), the feet (16.8%), the lumbar spine (14.7%), the shoulders (12.6%), and the groins (10.5%).

Among all injuries, 76 (82.6%) were considered overuse injuries, and 16 (17.4%) were acute injuries (Table 2). There was no significant difference in the onset mode between the different age categories (*P* = 0.73). The most common diagnosis was growth-related injuries (*n* = 38, 40%); these primarily affected the knee (15.8% had Osgood–Schlatter disease, and 9.5% had Sinding–Larsen disease, including five players who had both), followed by the foot (13.7% had Sever’s disease). Finally, one case of iliac spine osteochondrosis was reported (Table 3). The next most frequently represented conditions were shoulder tendinopathies (*n* = 11, 11.6%), chronic low back pain (*n* = 8, 8.4%), and hip impingement (*n* = 6, 6.3%). Among the acute injuries, 16 (94%) were non-contact, and only 1 was related to direct contact with the table. The most frequent diagnoses of acute injuries were lateral sprains (*n* = 5, 31%), acute low back pain (*n* = 5, 31%), and muscle injuries (*n* = 5, 31%: 2 hamstring injuries, one quadriceps injury, one adductor injury, and one pectoralis major injury).

4. Major injuries

**Table 3:** Injury diagnoses according to categories of age.

	Total *N* = 139	U-11 *N* = 33	U-13 *N* = 40	U-15 *N* = 48	U-19 *N* = 18	*P*
Upper limbs, *n*(%)	13 (13.7)	0	1	9	3	0.06
- Shoulder tendinopathy	11 (11.6)	0	0	8	3	
- Pectoralis major muscle injury	1 (1.1)	0	0	1	0	
- Epicondylitis	1 (1.1)	0	1	0	0	
Lower limbs, *n*(%)	68 (71.6)	13	24	24	7	
- Hip adductor tendinopathy	3 (3.2)	0	0	1	2	
- Hip impingement	6 (6.3)	0	1	3	2	
- Thigh muscle injury	4 (4.2)	0	1	2	1	
- Osteochondrosis	38 (40.0)	9	17	11	1	
- Iliac spine	1 (1.1)	0	1	0	0	
- Sinding–Larsen	9 (9.5)	2	4	3	0	
- Osgood–Schlatter	15 (15.8)	0	8	6	1	
- Sever	13 (13.7)	7	4	2	0	
- Patellofemoral syndrome	4 (4.2)	0	1	3	0	
- Medial knee pain	1 (1.1)	0	1	0	0	
- Periostitis	1 (1.1)	0	0	1	0	
- Chronic ankle pain	2 (2.1)	1	1	0	0	
- Ankle sprain	5 (5.3)	2	1	2	0	
- Fibular tendinopathy	1 (1.1)	0	0	1	0	
- Hallux fracture	1 (1.1)	0	1	0	0	
- Metatarsalgia	2 (2.1)	1	0	0	1	
Spine, *n*(%)	14 (14.7)	1	6	4	3	
- Acute low back pain	5 (5.3)	0	4	1	0	
- Chronic low back pain	8 (8.4)	1	1	3	3	
- Coccydynia	1 (1.1)	0	1	0	0	

During the follow-up, 12 injuries (12.6% of all injuries reported) resulted in more than 28 days off from sport (Table 2). The incidence of major injuries was 0.1/athlete/season and 0.2 injuries/1000 hours of exposure. There was no significant difference between the different age categories (*P* = 0.93). The longest periods of time loss were due to osteochondroses (two cases of Sever’s disease, two cases of Osgood–Schlatter disease, and one case of iliac spine osteochondrosis) (50% of major injuries), tendinopathies (rotator cuff and hip adductors) (17%), spine conditions (low back pain and coccydynia) (17%), one case of hip impingement, and one hallux fracture.

5. Injuries according to sex

Among all the players, 52 were females (37.4%), and 87 were males (62.6%). Both sexes exhibited comparable characteristics, particularly in terms of age (13.4 ± 2.2 years old vs. 13.3 ± 2.1 years old, *P* = 0.6) and number of training hours (13.9 ± 4.3 hours vs. 14.8 ± 4.1 hours, *P* = 0.22), height (157.9 ± 10.1 cm vs. 159.7 ± 14.4 cm, *P* = 0.43), and weight (49.6 ± 11.8 kg vs. 48.7 ± 12.9 kg, *P* = 0.68). Only BMI presented a significant difference (19.6 ± 2.9 vs. 18.7 ± 2.2, *P* = 0.04). A total of 44 injuries were reported among female athletes (i.e., 0.8 injuries/athlete/season and 1.6 injuries/1000 hours of exposure) and 51 among male athletes (i.e., 0.6 injuries/athlete/season and 1.0 injury/1,000 hours of exposure) (Table 4). No significant differences were observed between sexes when considering the overall injury incidence (*P* = 0.35). Similarly, no significant differences were found regarding the mode of onset (*P* = 0.99), the occurrence of time loss (*P* = 0.39), or its duration (*P* = 0.45). Injury location also did not differ significantly between male and female athletes (*P* = 0.34). The various injury diagnoses by sex are presented in Table 5.

**Table 4:** Injuries characteristics according to sex.

	Total *N* = 95	Male *N* = 51	Female *N* = 44	*P*
Injuries/athletes, *n*	0.7	0.6	0.8	0.35
Injuries/injured athletes, *n*	1.3	1.3	1.3	1
Mode of onset^a^				
*Gradual, n (%)*	76 (82.6)	40 (78.4)	36 (81.8)	0.99
*Sudden, n (%)*	16 (17.4)	9 (17.6)	7 (15.9)	
Time loss injuries, *n* (%)^b^	34 (35.8)	16 (31.4)	18 (40.9)	
≤7 days	10	6	4	0.45
8–28 days	12	4	8	
>28 days	12	6	6	

ᵃMissing data: 3.

ᵇMissing data: 9.

**Table 5:** Diagnoses of injuries according to sex.

	Total *N* = 95	Male *N* = 51	Female *N* = 44	*P*
Upper limbs, *n*(%)	13 (13.7)	7 (13.7)	6 (13.6)	0.34
- Shoulder tendinopathy	11 (11.6)	5 (9.8)	6 (13.6)	
- Pectoralis major muscle injury	1 (1.1)	1 (2.0)	0 (0.0)	
- Epicondylitis	1 (1.1)	1 (2.0)	0 (0.0)	
Lower limbs, *n*(%)	68 (71.6)	39 (76.5)	29 (65.9)	
- Hip adductor tendinopathy	3 (3.2)	2 (3.9)	1 (2.3)	
- Hip impingement	6 (6.3)	1 (2.0)	5 (11.4)	
- Thigh muscle injury	4 (4.2)	2 (3.9)	2 (4.5)	
- Osteochondrosis	38 (40.0)	26 (51.0)	12 (27.3)	
Iliac spine	1 (1.1)	0 (0.0)	1 (2.3)	
Sinding–Larsen	9 (9.5)	6 (11.8)	3 (6.8)	
Osgood–Schlatter	15 (15.8)	9 (17.6)	6 (13.6)	
Sever	13 (13.7)	11 (21.6)	2 (4.5)	
- Femoropatellar syndrome	4 (4.2)	2 (3.9)	2 (4.5)	
- Medial knee pain	1 (1.1)	1 (2.0)	0 (0.0)	
- Periostitis	1 (1.1)	1 (2.0)	0 (0.0)	
- Chronic ankle pain	2 (2.1)	0 (0.0)	2 (4.5)	
- Ankle sprain	5 (5.3)	3 (5.9)	2 (4.5)	
- Fibular tendinopathy	1 (1.1)	0 (0.0)	1 (2.3)	
- Hallux fracture	1 (1.1)	1 (2.01	0 (0.0)	
- Metatarsalgia	2 (2.1)	0 (0.0)	2 (4.5)	
Spine, *n*(%)	14 (14.7)	5 (9.8)	9 (20.5)	
- Acute low back pain	5 (5.3)	2 (3.9)	3 (6.8)	
- Chronic low back pain	8 (8.4)	2 (3.9)	6 (13.6)	
- Coccydynia	1 (1.1)	1 (2.0)	0 (0.0)	

## Discussion

This study was conducted to better understand and assess the risk of musculoskeletal injuries among young table tennis players. To our knowledge, this is the first study investigating the epidemiology of training-related injuries in young table tennis athletes with such a large sample size and detailed information on etiological diagnoses.

Overall, 95 injuries were reported among the 139 table tennis athletes followed-up, indicating a relatively low incidence rate of 1.2 injuries per 1000 hours of training. In comparison, the injury rate is lower than that reported in other studies focusing on adolescent table tennis players, although injury incidence remains relatively low across all studies. Indeed, Pullinger *et al*^[^9^]^ showed an incidence rate of 8.3 injuries per 1000 hours of exposure, and Rejeb *et al*^[^8^]^ reported an incidence rate of 4.5 injuries per 1000 hours of exposure. This difference may be explained by the limited number of athletes included in these two studies (8 and 20 table tennis players, respectively), compared to the larger sample size in our study. Regarding studies conducted on adult athletes, very few report injury incidence rates based on exposure time. Furthermore, the significant heterogeneity in data collection and analysis methods makes comparisons with our results difficult. Indeed, a 2022 meta-analysis^[^7^]^ highlighted a moderate-to-high risk of bias in approximately 93% of cases, indicating that very few studies are of high methodological quality. In comparison, the frequency of injuries seems to be higher during competitions, with an incidence rate of 10 injuries per 1000 hours of exposure^[^6^]^. A similar trend has been observed in several other studies, reporting that around 60% of injuries occur during competitions^[^14,15^]^.

In this study, most of the injuries were not serious. Only 35.8% of the injuries resulted in time loss, and 12.6% were major injuries that required more than 28 days off from sport (i.e., 0.2 injuries/1000 hours of exposure). The rate of major injuries was probably slightly overestimated, as two players stopped playing table tennis entirely following their injury, although this decision may not have been directly related to the injury itself. Similar findings have been reported in other studies, whether among adults competing at regional or national levels (32% of time loss and 6% of major injuries)^[^16^]^, or among youth athletes participating in international competitions (25% of time loss)^[^17^]^. The only study focusing on training-related injuries in young players^[^9^]^ reported a higher rate of sports discontinuation (53%), but none lasting longer than 28 days. Once again, this may be explained by the very small sample size of that study. It is interesting to note that growth-related injuries led to the longest periods of time loss (12 and 16 days), which is consistent with our findings (in our study, 50% of major injuries were due to growth-related injuries).

As reported in other studies^[^7,16^]^, the majority of injuries were overuse injuries (82.6% of cases). This can be attributed to the specific demands of table tennis, a sport characterized by a succession of rapid, powerful, and explosive movements required to return the ball. The repetitive nature of these actions – whether during training sessions, warm-ups, or competitive matches – leads to microtrauma. When recovery periods are insufficient, the body’s capacity for tissue repair may be exceeded, resulting in chronic musculoskeletal injuries. Identifying the types of these overuse injuries and associating them with specific technical movements is therefore essential. Such knowledge would allow better structuring of training programs, physical conditioning, and rest periods to prevent athletes from reaching a point where physiological repair mechanisms are overwhelmed. It is noteworthy that only Lesguer^[^18^]^ reported a higher incidence of acute traumatic injuries compared to chronic ones. However, this finding may be explained by the study’s methodology, which relied on a retrospective questionnaire sent to athletes, asking them to report all injuries sustained throughout their careers. This approach is subject to significant recall bias, potentially leading to an over-reporting of acute injuries and an under-reporting of chronic conditions that did not require cessation of athletic activity.

In our study, similar injury characteristics were observed regardless of age or sex, particularly in terms of frequency, mechanism of occurrence, and time off from sport. However, there was a trend toward a lower proportion of lower limb injuries in older age categories, even though no statistically significant difference was identified. This result can be explained by a lack of statistical power due to an insufficient sample size. Similarly, the number of injuries was insufficient to allow a detailed comparison of the different etiological diagnoses according to age categories or to identify specific injury risk factors. The number of training hours was significantly higher only for juniors compared to Benjamins, and no significant differences were found between these two groups in terms of characteristic injuries. Regarding sex, the groups were comparable, and sex did not appear to have an impact on either injury risks or injury locations. Although few studies have investigated this subject^[^14,16,18^]^, none has found a difference in injury frequency, thereby supporting our findings. Other characteristics of the population – such as athletes’ handedness, playing style, or type of racket – were not available for a sufficient number of participants and therefore could not be included in the analyses. Further studies with larger sample sizes and a specific focus on injury risk factors are therefore needed.

Knee was the most frequent location in our study (30.5%), and knee injuries were mostly represented by osteochondroses (82.8%) and patellofemoral syndrome (13.8%). We found a higher rate of knee injury incidence than in most other studies, which may be explained by differences in the populations analyzed. Indeed, growth-related osteochondroses are common in young individuals, whereas these conditions do not occur in adults. The knee still remains one of the most commonly affected joints in the literature, after the shoulder^[^15,19^]^. The main injuries reported in our study were osteochondroses (40%), affecting only the lower limb (63.2% the knee, 34.2% the foot, and 2.6% the pelvis). This is consistent with findings from other studies on adolescent table tennis players^[^8,9^]^. Although osteochondroses are well-known conditions and have been studied for a long time, their etiopathogenesis is not yet fully understood. Nevertheless, several risk factors have been identified^[^20,21^]^, such as age (growth period), intense physical activity, history of osteochondroses, muscle stiffness (e.g., quadriceps in Osgood–Schlatter disease, gastrocnemius-soleus complex in Sever’s disease), and anatomical factors (such as external tibial torsion in Osgood–Schlatter disease). While evidence on effective prevention strategies remains limited, studies emphasize that young athletes should observe minimum periods of active rest, engage in appropriate physical training, and vary sport-specific tasks. Stretching is also encouraged, particularly in at-risk populations.

The second most frequent injury affected the shoulder: rotator cuff tendinopathy (11.6%). It is the most commonly reported injury in most other studies^[^14,15,19,22^]^. The shoulder is particularly solicited during forehand and backhand topspin strokes. The forehand topspin is the most frequently used and most offensive technical stroke in table tennis^[^3^]^. It requires rapid and explosive movements during which both muscle contraction force and joint range of motion are highly significant. During the striking phase of a forehand topspin, internal rotation, shoulder flexion, and adduction occur^[^23^]^, and the abduction/adduction range of motion may be even greater when the stroke is performed against a backspin ball^[^24^]^. Whether in forehand or backhand strokes, racket speed is strongly associated with shoulder movement. A large number of repetitions of these movements over many hours of practice can lead to an imbalance between the internal and external rotator muscles, as well as between the scapular stabilizers, thereby compromising the dynamic functional stability of the glenohumeral joint. Meghdadi *et al*, using electromyography, observed a dysfunction of shoulder muscles in table tennis players with impingement syndrome. This dysfunction is characterized by a disorder in the activation and recruitment sequence of shoulder muscles and a significant reduction in the activity level of the serratus anterior and supraspinatus muscles. This contributes to an abnormal movement of the scapula and dynamic shoulder instability^[^25^]^. Moreover, it has been shown that significant differences exist in internal rotation range of motion between the two sides in table tennis players. Zhang *et al* found that this deficit is associated with self-reported shoulder pain in the past year, with a cutoff point for the difference in glenohumeral internal rotation range of motion of 17.9 degrees in the supine position^[^26^]^. These values could be used clinically as warning thresholds to identify a potential risk of injury or pain related to a mobility deficit.

In our study, spine conditions were common and were responsible for chronic and acute injuries (8.4% of injuries were chronic low back pain, and 5.3% were acute low back pain). This is consistent with what has already been highlighted in the literature, with a frequency among all injuries ranging from 10% to 25% in most studies^[^9,14,19,27^]^. The execution of technical strokes in table tennis relies on an open kinetic chain, whereby mechanical energy is transferred from proximal segments (i.e., lower limbs, trunk, and shoulders) to distal segments (i.e., elbow, forearm, hand, and racket) throughout the stroke. This energy transfer is particularly dependent on axial trunk rotation^[^4^]^. Additionally, lateral trunk flexion movements are performed depending on the targeted area of the opponent’s side of the table (down-the-line or cross-court stroke)^[^28^]^. Moreover, between strokes, players must remain in a state of readiness to respond to the opponent’s actions, referred to as the ready position. Studies have demonstrated that adopting this position leads to a more than 10-fold increase in anterior trunk inclination compared to a standard upright posture. Significant alterations are also observed in spinal curvature angles: a threefold increase in upper thoracic and lumbosacral curvature and a twofold increase in thoracolumbar curvature. These changes indicate an accentuation of thoracic kyphosis and lumbar lordosis. Consequently, trunk asymmetry in the neutral posture has been identified in table tennis players compared to non-players^[^29^]^. This morphological asymmetry may predispose athletes to musculoskeletal pain and injury. As such, implementing targeted physical conditioning programs aimed at reducing these asymmetries may prove beneficial. However, current literature on this topic remains limited, warranting further investigation^[^30^]^. Finally, standard preventive strategies for spinal pathologies – such as strengthening the abdomino-pelvic musculature and improving the flexibility of sub-pelvic structures – should be systematically employed to mitigate the risk of such conditions.

Some French studies in recent years have focused on hip conditions, as their prevalence has increased at the highest level of competition. This has led to the implementation of a specific screening examination for hip pain during the mandatory medical monitoring of elite table tennis athletes^[^31^]^. Our study indeed revealed a relatively high prevalence of hip impingement (6.3%), even at a young age. According to the literature, regular and high-intensity sports practice (from three training sessions per week in addition to weekend competitions) during adolescence – particularly in sports that place significant demands on the hip joint, such as ice-hockey, basketball, and soccer – is associated with an increased risk of femoral head-neck deformity associated with femoroacetabular impingement^[^32^]^. This risk is reported to be 1.9–8 times higher compared to non-athletes. In table tennis, the hip joint plays a central role by connecting the lower to the upper limbs and transferring energy from the ankle and knee to the upper body through internal rotation and extension on the racket-holding side during the forward stroke phase^[^33^]^. This internal rotation is strongly correlated with racket speed at the time of ball impact. Segmental contributions from the racket-side hip are most significant during pivot movements and lateral steps. Hip impingement is a source of disabling pain. In our study, half of the cases resulted in time loss, including one instance of more than 28 days off. Furthermore, it can lead to intra-articular damage and even require surgical intervention. It is therefore crucial to implement preventive strategies as part of the training routines of young table tennis players engaged in intensive practice.

Our results confirm the importance of strategies to prevent injuries in young table tennis players. As mentioned in other sports, structured warm-up programs are particularly effective in reducing injury incidence^[^34^]^. These programs should also be complemented by specific supervised strengthening exercises tailored to the biological maturity of the players. Systematic screenings, such as those conducted in our study, may help detect injuries before they become too serious, which might explain our lower incidence rate compared to existing literature. In the case of injuries, appropriate rest should be recommended to prevent any worsening of the condition, and the return to sport should be structured, progressive, and supervised by coaches in accordance with medical advice.

Our study is one of the largest cohort studies on musculoskeletal injuries among table tennis athletes. One of the main strengths is the systematic annual evaluation and the possibility of additional consultations in case of injury, conducted by an experienced physician. This specific organization helps reduce the risk of missing data, although we cannot completely exclude the possibility that some athletes may have consulted other practitioners. We only considered injuries that required medical evaluation, which may underestimate the number of minor injuries and, therefore, the overall injury incidence rate. However, this method of assessment by physicians specializing in sports medicine increases the reliability of the results and allows for more accurate medical diagnoses. One of the main limitations of the study is its retrospective nature, which carries an inherent risk of measurement bias. As previously mentioned, the sample size was still insufficient for certain analyses, and a potential loss of statistical power could explain some of the results. Larger-scale, prospective studies with more participants are, therefore, still essential.

## Conclusion

This study found an injury rate of musculoskeletal injuries of 1.2 injuries per 1000 hours of training or 0.7 injuries per athlete per season. Most of the injuries were chronic overuse injuries, and the most frequent diagnoses were osteochondroses, shoulder tendinopathy, chronic low back pain, and hip impingement. A thorough understanding of the nature of injuries and their mechanisms of occurrence is essential to improve medical management as well as the implementation of effective preventive measures. Further multicenter research is needed to confirm these findings and identify potential risk factors.

## Data Availability

The data are available upon reasonable request to the corresponding author. The dataset used and analyzed during the current study is available from the corresponding author upon reasonable request.

## Research registration unique identifying number (UIN)

This is a retrospective study.
